# School Readiness in 4-Year-Old Very Preterm Children

**DOI:** 10.3390/children9030323

**Published:** 2022-03-01

**Authors:** H. Gerry Taylor, Daphne M. Vrantsidis, Mary Lauren Neel, Rebekah Benkart, Tyler A. Busch, Aryanne de Silva, Shivika Udaipuria, Nathalie L. Maitre

**Affiliations:** 1 Abigail Wexner Research Institute, Nationwide Children’s Hospital, Columbus, OH 43215, USA; daphne.vrantsidis@nationwidechildrens.org (D.M.V.); marylauren.neel@nationwidechildrens.org (M.L.N.); rebekah.benkart@nationwidechildrens.org (R.B.); tyler.busch@nationwidechildrens.org (T.A.B.); aryanne.desilva@nationwidechildrens.org (A.d.S.); 2 Department of Pediatrics, The Ohio State University, Columbus, OH 43210, USA; 3 Department of Epidemiology, Rollins School of Public Health, Emory University, Atlanta, GA 30322, USA; u.shivika@emory.edu; 4 Department of Pediatrics, Emory University, Atlanta, GA 30322, USA; nathalie.linda.maitre@emory.edu

**Keywords:** school readiness, very preterm birth, preschool children

## Abstract

The aims of this study were to identify the aspects of school readiness that best distinguish very preterm (VPT) preschoolers from full-term (FT) controls, determine the extent to which readiness problems in the VPT group reflected global cognitive weaknesses or more specific deficits, and identify distinct profiles of readiness problems. Fifty-three VPT (gestational age ≤ 30 weeks) 4-year-olds were compared to 38 FT (gestational age ≥ 37 weeks) controls on measures of global cognitive ability, executive function, motor skills, early literacy and numeracy, and psychosocial functioning. Latent class analysis (LCA) was also conducted to identify individual readiness profiles. The VPT group had the most pronounced difficulties on tests of spatial and nonverbal cognitive abilities, executive function, motor skills, phonological processing, and numeracy. The VPT group also had sex-related difficulties in processing speed, social functioning, and emotion regulation. These differences were evident in analyses of both continuous scores and rates of deficits. The VPT group’s difficulties in motor skills, and VPT females’ difficulties in social functioning and emotion regulation, were evident even when controlling for global cognitive ability. LCA suggested four profiles of readiness, with the majority of the VPT group assigned to profiles characterized by relative weaknesses in either cognitive abilities or psychosocial functioning or by more global readiness problems. The findings support the need to evaluate multiple aspects of school readiness in VPT preschoolers and inform efforts to design more targeted early educational interventions.

## 1. Introduction

Very preterm (VPT) children, typically defined as those born at a gestational age (GA) < 32 weeks, are at higher risk for deficits in academic achievement than full-term (FT) children [1,2]. These problems persist across the school-age years, are inversely related to the degree of prematurity at birth, and are preceded by earlier developmental delays [1,2,3,4,5,6].

According to the American Academy of Pediatrics [7] and an updated technical report [8], school readiness covers a broad range of characteristics conducive to learning, including health and physical/motor development, social–emotional adjustment, approaches to learning as exemplified by motivation and self-control, communication skills, and general knowledge (including knowledge of letters and numbers) and cognition. More recently, the concept of school readiness has been expanded to include factors outside the child, such as family, school, and community supports, that can foster readiness if present or hamper it if absent [8,9]. In an Australian sample, a delay in at least one of these characteristics was observed for 44% of VPT 5-year-olds compared to 16% of FT controls [10]. Researchers in New Zealand documented generalized delays in school readiness skills at age 4 years in VPT children compared to FT controls [4]. In the latter study, delays in multiple areas of readiness were three times more common in the VPT group compared to the FT group and predicted later deficits in academic achievement at ages 6 and 9 years. Other studies provide additional evidence for deficits in cognitive and emotional self-regulation in VPT preschoolers and associations of these deficits with learning problems [11,12,13,14,15,16,17,18,19].

Findings indicating that some VPT children have selective deficits in executive function, motor skills, and mathematics, while others appear free of any discernable impairments, raises the possibility of individual variability in school readiness profiles [20,21,22]. Individual variability is also supported by findings that suggest different profiles of school readiness skills in community samples of children [9,23,24] and of behavior problems in extremely preterm (GA < 28 weeks) children [25]. Using cluster or latent class analysis (LCA), these studies identified profiles suggesting that some children have pervasive problems in readiness skills or behavior while others have more selective deficits or are functioning well in all areas.

Findings from two more recent studies that employed LCA provide additional support for different profiles of cognitive, motor, and behavior outcomes at early school age. In following a large French sample of VPT children (GA < 32 weeks) to age 5.5 years for the EPIPAGE-2 Study, Twilhaar and colleagues [26] identified four profiles of cognitive, motor, and psychosocial characteristics. One profile was characterized by outcomes that were comparable to those of a FT sample, a second by more difficulties in psychosocial adjustment than in cognitive and motor skills, a third by more problems in cognitive and motor abilities than in psychosocial adjustment, and a fourth by difficulties in all areas relative to the FT children. A study of another sample of 5-year-old VPT children (GA < 30 weeks) and a FT comparison group also identified four profiles [27]. One profile was characterized by age-typical cognitive, motor, language, and psychosocial characteristics, a second by mild cognitive, motor, and language deficits in the context of more typical psychiatric ratings, a third by parent reports of pronounced psychosocial problems, and a fourth by teacher reports of relatively severe symptoms of attention problems and autism spectrum disorder.

However, evidence of individual differences in profiles of school readiness among VPT preschoolers is limited. The relationship between cognitive or motor deficits and behavior problems in these children is also unclear. Although cognitive impairments are associated with behavior problems in VPT cohorts [28,29,30], further research is needed to examine the possibility of relatively isolated deficits in behavior or performance-based measures of cognitive and motor functioning in preschoolers.

The objectives of this study were to enhance knowledge of the effects of VPT birth on school readiness in three ways. First, we compared groups of VPT preschoolers and FT controls on multiple measures of readiness to identify the breadth and magnitude of readiness problems in the VPT group and to confirm that our cohort had deficits similar to those observed in seminal studies of VPT preschoolers [8,9,10,13,17,20,24,25,26]. The two groups were compared on both continuous measures of readiness and rates of deficits on these measures. A second objective was to examine the extent to which problems in different readiness domains varied independently of one another. Thirdly, we conducted LCA to examine evidence for distinct profiles of readiness and their association with group membership.

Information regarding the type and magnitude of VPT preschoolers’ deficits in readiness will help in targeting interventions for these children’s most pressing needs. Evidence for distinct types or profiles of deficits will also contribute to an improved understanding of individual variability in readiness problems and will guide in creating assessments of readiness that are sensitive to the full range of readiness problems. Based on research on the consequences of VPT birth in young children, we hypothesized that VPT preschoolers would differ from FT controls on a broad array of readiness measures, but that the magnitude of these differences would vary across measures. Consistent with past evidence for individual variability in outcomes of VPT birth, we also anticipated multiple types and patterns of group differences.

## 2. Materials and Methods

### 2.1. Sample

As described in studies of resilience and positive adaptation in VPT preschoolers [31,32], VPT children were recruited by contacting families of children treated in the Follow-Up program for a network of neonatal intensive care units affiliated with Nationwide Children’s Hospital in Columbus, Ohio USA. To examine outcomes in a higher-risk VPT preschool sample, only children with GA ≤ 30 weeks were included. Children had to be 4 years of age at the time of recruitment. Children from non-English speaking families and those with genetic abnormalities known to affect cognition were excluded. Given our interests in examining performance-based measures of readiness, we also excluded children with severe sensory impairment. To capture as many children as possible within the recruitment period (September 2018 to June 2019), VPT preschoolers were recruited according to age (older to younger) from a larger hospital-based sample of VPT children. The VPT group comprised 53 of 60 eligible children. Seven children were not seen because of missed or canceled appointments. Comparison of these 7 children to the participants failed to reveal significant differences in GA, age at assessment, or sex (ps > 0.05). FT controls included 38 children recruited at 4 years of age during the same period as the VPT group. Recruitment was from flyers emailed to hospital employees who had volunteered for research.

Table 1 summarizes the birth and demographic characteristics of the two groups. The VPT group had a significantly lower z-score composite of SES (zSES) than the FT group (*p* < 0.001), with zSES defined as the mean of sample z-scores for measures of caregiver education, occupation, and census-based median family income [33]. The groups did not differ significantly in age at assessment, sex, or race.

### 2.2. Procedures and Measures

One group of examiners administered a battery of readiness tests to the children while different examiners supervised caregivers’ completion of child behavior rating scales. Children’s birth status was not shared with the child examiners. Child testing was completed in four half-hour sessions spanning approximately three hours, with breaks between the sessions. Tasks were administered in one of three orders, with children assigned randomly to an order. The study was approved by the hospital Institutional Review Board and caregivers provided informed consent before participation.

Measures of readiness were divided into the five domains listed in Table 2: global cognitive ability, executive function and processing speed, motor skills, early literacy and numeracy skills, and behavior problems. Because of the extensive measures of behavior problems provided by the Conners Early Childhood-Parent questionnaire (Conners EC-P) [36], only scores for total behavior problems and for subdomains previously shown to be adversely affected by preterm birth were considered [37]. Scores were age-adjusted for measures with normative standards. Adjustments were made for chronological rather than corrected age given that school readiness is typically evaluated based on age since birth. Age norms were not available for the Zoo Go No Go Test [38] and Emotion Regulation Checklist (ERC) [39]; thus, raw scores were used in analyses of these measures. Table 2 includes brief descriptions of the traits assessed by each measure and citations that document acceptable levels of reliability and validity. Test validity is further documented by previous research with young VPT or very low birth weight (<1500 g) children using these or similar tests [11,12,40,41,42,43].

### 2.3. Analysis

Mixed model analyses were used to examine group differences. Covariates in these and other analyses included sex and zSES. A random effect accounted for dependence between sibling participants. The relative magnitudes of group differences were assessed in terms of effect size (ES).

The possibility that the VPT and FT groups differed along multiple dimensions of school readiness was examined in two ways. First, the above-noted mixed model analyses were repeated controlling for the Differential Ability Scales, 2nd Edition General Conceptual Ability (DAS-II GCA) [44] to determine if group differences could be attributed to differences in global cognitive ability. Second, a general estimating equation (GEE) analysis was conducted to examine the possibility of multiple independent associations of readiness measures with group membership. Predictors in this analysis were limited to the one measure from each of the five domains that discriminated the groups with the largest ES.

Additional analyses using GEE were conducted to compare the groups (coded as FT = 0 and VPT = 1) on rates of deficits. Deficits were defined as scores ≥ 1SD below the mean on the performance measures and ERC Emotion Regulation, and scores ≥ 1SD above the mean on all ratings of behavior problems except for ERC Emotion Regulation. To determine if the groups differed in rates of deficits, the number of deficits was tallied across individuals, and the groups were compared on rates of two or more deficits. Although group differences in analyses using both mixed models and GEE were similar whether scores were adjusted for chronological age or for GA, only results from analysis of scores adjusted for chronological age are reported here.

Because of the descriptive nature of the study and limited sample size, an uncorrected *p*-level of <0.05 was applied to determine statistical significance. Information on the magnitude of effects (EFs) was provided by Cohen’s d for group comparisons on continuous measures and by odds ratios (and 95% confidence intervals) for results from GEEs. Small, medium and large effects from mixed models were defined as Cohen’s d’s of 0.2, 0.5, and 0.8, respectively [51]. Cohen’s d was calculated using the estimated marginal means and standard errors. These values adjust for the effects of the covariates (e.g., zSES) on the point estimates.

LCA was used to investigate individual differences in profiles of deficits. Variables included in these analyses were most of those listed in Table 2. Measures excluded from these analyses were the DAS-II GCA in order to focus on potential variations across profiles in the verbal, nonverbal, and spatial ability scores. Subscales of the Behavior Rating Inventory of Executive Function-Preschool (BRIEF-P) [50] and Movement Assessment Battery for Children, 2nd Edition (MABC-2) [47] were also excluded to limit the number of variables. The Children’s Test of Phonological Processing, 2nd Edition (CTOPP-2) [46] rapid naming tests were excluded because of missing data due to many children’s inability to consistently name the stimuli. Analyses were conducted using Mplus version 8 and SAS 9.4. For families with multiple participants, one child from each family was randomly selected to avoid biases related to family clustering, with preference given to children with the most complete data. Seventeen children (15 PT, 2 FT) were excluded on this basis, resulting in a total sample of 75 children for LCA (39 VPT, 36 FT).

LCA models were run with 1 to 5 latent classes. To determine the best-fitting model, each model was compared to the model with one fewer class. The best-fitting model was selected based on fit statistics and interpretability. Model fit was assessed using the Bayesian information criteria (BIC), Akaike information criteria (AIC), adjusted BIC, Vuong–Lo–Mendell-Rubin test, Lo–Mendell test, and bootstrap likelihood ratio test. Lower values on BIC, AIC and adjusted BIC indicated better model fit. A significant *p*-value on the Vuong–Lo–Mendell–Rubin test, Lo–Mendel-Rubin test, or bootstrap likelihood ratio test indicated a better fit for the less parsimonious model (e.g., the 2-class over the 1-class model). Once the best-fitting model was determined, it was used to classify children into their most likely class. Entropy was used to determine classification accuracy. The value of entropy ranges from 0 to 1, with higher values indicating better accuracy. Finally, analyses of variance were conducted to compare the children assigned to each class on the readiness measures, group membership, sex, and zSES.

## 3. Results

### 3.1. Group Differences in Continuous Measures of Readiness

The VPT group had significantly lower scores on all tests except the NIH Toolbox Dimensional Change Card Sorting Test [45] and all caregiver behavior ratings except the Conners EC-P Global Index, Inattention/Hyperactivity, and Anxiety scales, BRIEF-P Inhibitory Self-Control indices, and Flexibility indices, and ERC Lability/Negativity (see Table 3). Large ESs were found for DAS-II GCA, Nonverbal Ability, Spatial Ability, Recall of Digits-Forward, and Early Number Concepts; MABC-2 Total, Manual Dexterity, and Balance; and the Phonological Awareness subtest of the Test of Preschool Early Literacy (TOPEL) [48]. Analyses also revealed significant group × sex interactions for the CTOPP-2 Rapid Symbolic Naming, Conners EC-P Social Functioning, and ERC Emotion Regulation, as well as a significant group × zSES interaction for MABC-2 Aiming and Catching. VPT boys had lower CTOPP-2 Rapid Symbolic Naming scores than FT boys (*p* = 0.002; ES = −1.18). Compared to FT girls, VPT girls had higher Conners EC-P Social Functioning (i.e., worse social functioning) (*p* < 0.001, ES = 1.42) and lower ERC Emotion Regulation (*p* < 0.001, ES = 1.20).

Main effects for sex and zSES were significant in analyses of several of the measures (data not shown). Boys had higher scores than girls on DAS-II Recall of Digits-Forward and lower scores on the MABC-2 Total. Higher zSES was associated with higher scores on DAS-II Spatial Ability and TOPEL Print Knowledge and with lower ratings of behavior problems on the Conners EC-P Global Index and Inattention/Hyperactivity; BRIEF GEC, Inhibitory Self-Control, Flexibility, and Emergent Metacognition; and ERC Lability/Negativity.

Even when adjusting for the DAS-II GCA, differences remained significant for MABC-2 Total (*p* = 0.002, ES = 0.74) and Manual Dexterity (*p* = 0.015, ES = 0.60). Consistent with results from analyses that did not adjust for the DAS-II GCA, these analyses also revealed significant group × sex interactions for Conners EC-P Social Functioning (*p* < 0.001) and ERC Emotion Regulation (*p* = 0.003). VPT girls had significantly higher ratings of problems than FT girls on Conners EC-P Social Functioning (*p* < 0.001, ES = 1.21) and lower ratings on ERC Emotion Regulation (*p* = 0.003, ES = −1.11). Group differences in these measures were not significant for boys.

In the GEE analysis that included the measure from each domain with the largest ES as predictors of group membership (DAS-II GCA, Recall of Digits-Forward, and Early Number Concepts; MABC-2 Total; and BRIEF-P Emergent Metacognition Index), the MABC-2 Total was the only measure significantly associated with group independently of the other predictors, beta (standard error) = 0.35 (0.14), *p* = 0.011.

### 3.2. Group Differences in Rates of Deficits

Similar to results from mixed model analyses, odds of deficits were significantly higher for the VPT group than for the FT group on most of the measures (see Table 4). Rates of deficits on the performance tests for which group differences were significant ranged from 18% to 74% for the VPT group compared to 0% to 26% for the FT group. A significantly higher proportion of the VPT group also had deficits on Conners EC-P Social Functioning, BRIEF-P GEC, Inhibitory Self-Control, and Emergent Metacognition. Rates of deficits on these measures ranged from 32% to 57% for the VPT group compared to 3% to 16% for the FT group. Deficits in multiple readiness measures were also significantly higher in the VPT group compared to the FT group (83% versus 32%).

### 3.3. Readiness Profiles

**Figure 1:**
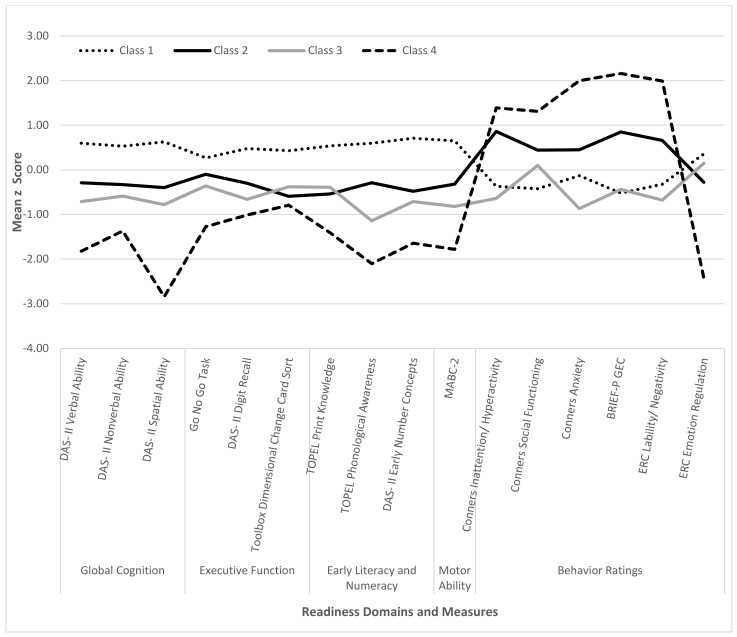
Latent class differences in mean z scores for readiness measures.

Model fit statistics were better for the 4- and 5-class models relative to the 1-, 2- and 3-class models (Table 5), thus the 1- to 3-class models were rejected. The 5-class model compared less favorably to the 4-class model based on Entropy and BIC. The 4-class model, shown in Figure 1, was selected as it was more interpretable and resulted in lower BIC indices and higher entropy relative to the 5-class model.

The 37 children assigned to latent class 1 had the best readiness outcomes, with scores on performance tests and behavior ratings falling well within the average range. Only eight VPT children were in this class (22% of class). Scores on all performance tests were lower for the 20 participants in latent class 2 than for those in class 1. Fourteen of these children (70%) were from the VPT group. Although latent class 2 participants had higher test scores than those in latent classes 3 or 4, they had more behavior problems than the 14 children in latent class 3, of whom 13 (93%) were from the VPT group. The four participants assigned to latent class 4, all from the VPT group, had the worst outcomes on all readiness measures.

Although group separation is optimized in LCA, support for these interpretations was provided by pairwise comparisons of readiness scores for the four latent classes. Specifically: (1) each of the four latent classes differed significantly from the other classes on the DAS-II Verbal and Spatial Ability and Conners EC-P Anxiety; (2) class 1 had significantly higher test scores than classes 2 and 3 on DAS-II Verbal, Nonverbal, and Spatial composites, DAS-II Recalling of Digits-Forward, TOPEL Print Knowledge and Phonological Awareness, DAS-II Early Number Concepts, and MABC-2, as well as significantly lower ratings on Conners EP-C Anxiety; and (3) class 2 had higher scores than class 3 on TOPEL Phonological Awareness but significantly higher ratings of behavior problems on Conners EC-P Inattention/Hyperactivity and Anxiety, BRIEF-P GEC, and ERC Lability/Negativity. The four latent classes also differed significantly in group membership (significantly higher proportion of the FT group in class 1 compared to the class 3), zSES (significantly higher in class 1 compared to class 2), and GA (significantly higher in class 1 compared to classes 1, 2, and 3).

## 4. Discussion

This study compared 4-year-old VPT children to FT controls on a wide range of measures of school readiness to identify measures of readiness on which the VPT group had the greatest difficulty and to examine variations in readiness problems. The VPT group performed most poorly relative to the FT group on tests of spatial ability, verbal working memory, motor performance, phonological processing, and number skills. Problems in psychosocial functioning that distinguished the VPT group from FT controls were in areas of executive function, and, at least for females, in emotion regulation and social functioning, although the ESs corresponding to these differences were not as large as those for group differences on the aforementioned performance measures. Group differences in rates of deficits were also the most pronounced for the performance measures. For example, nearly one-third of VPT children had deficits in global cognition compared to none of the FT preschoolers. Deficits similar to those observed in this study are reported in previous studies of both VPT preschoolers [4,10,11,17,22,52] and school-age VPT children [16,33,37,53,54,55,56,57]. The present study adds to this literature by assessing the variability of impairment displayed by VPT preschoolers across a range of readiness measures.

Although differences between the VPT and FT group were largely independent of sex and zSES in this and other studies [18], deficits in CTOPP-2 Rapid Symbolic Naming were found only for VPT boys, and more caregiver-rated problems on the Conners EC-P Social Functioning and weakness in ERC Emotion Regulation were evident only for VPT girls. Despite inconsistencies in reports of sex-related differences in the consequences of VPT birth, some previous research supports the possibility of more pronounced effects for males than for females [58]. We are unaware of evidence to suggest that females are more vulnerable than males to adverse socioemotional consequences of VPT birth. Potential explanations include gender differences in early socialization processes [59] or gender-related biases in parent ratings of socioemotional functioning [60]. We also found that differences in favor of the FT group on motor skills, as assessed by the MABC-2 Total, were more pronounced among children with lower zSES. The moderating effect of SES on group differences in motor ability is similar to findings suggesting more adverse effects of maternal insensitivity on academic achievement for low birth weight children compared to normal birth weight youth [61], and raises the possibility that readiness problems in VPT preschoolers may be exacerbated by environmental disadvantage. The latter finding is also consistent with evidence for a greater intervention effect on motor outcome for VPT children at higher social risk [62]. However, support from our study for sex- and zSES-related differences in the effects of VPT birth were limited to these measures. In view of the lack of more consistent evidence for these factors as moderators of VPT outcomes from this study or past research, caution is advised in interpreting these findings pending larger-scale studies of VPT preschoolers’ school readiness.

Most of the group differences in school readiness were not significant when adjusting for the DAS-II GCA, suggesting that problems in school readiness for many VPT preschoolers are associated with weaknesses in global cognitive ability. The finding of higher rates of multiple deficits in the VPT preschoolers compared to the FT group also points to pervasive problems in readiness in many VPT children. The results confirm previous findings of pervasive developmental deficits in VPT cohorts, as well as observations of associations between cognitive weaknesses and behavior problems in these children [28,29].

However, other findings from this study indicate that some children may have more selective problems in readiness. To begin with, even controlling for the DAS-II GCA, the VPT group relative to FT controls performed more poorly on measures of motor skills, and VPT girls were rated by caregivers as having more problems in social functioning and lower levels of emotion regulation relative to FT girls. Second, in GEE analysis that included a set of the most discriminating readiness measures from each of the five domains as predictors of group membership, motor ability discriminated the groups independently of other measures of readiness. These results underscore motor skills as a critical domain in assessment of readiness [4,10,63,64], and suggest that, at least for females, some socio-emotional aspects of school readiness may be affected independently of their cognitive competencies.

Third, findings from the LCA provided support for four individual patterns of readiness competencies. Classes 1 and 4, respectively, identified preschoolers with either strengths or weaknesses across all readiness domains. The other two classes were characterized by scores on performance tests that fell between those of classes 1 and 4 but differed in their profiles of relative strengths and weaknesses. Relative to the children assigned to class 3, those in class 2 performed at somewhat higher levels on tests of cognitive and motor skills but also had higher caregiver ratings of behavior problems. These findings are consistent with research suggesting that some VPT children display age-typical outcomes across multiple developmental domains, while others have either selective or generalized impairments [21,22,25,26,27,31,41]. Research examining readiness profiles in community samples of kindergarteners or first graders reveals similar findings [9,23,24]. As was evident in the present sample, these studies identified subgroups of children with uniformly positive or negative outcomes across readiness domains, as well as subgroups showing dissociations between cognitive and behavioral aspects of readiness. Evidence for latent classes comprising both VPT and FT preschoolers (i.e., classes 1–3) is also consistent with results from prior LCAs that have classified children from both these groups into the same behavioral profiles [25,65].

These findings need to be considered in the context of several study limitations. First, because of the numerous school readiness measures examined, the relatively small sample, and the lack of correction for multiple comparisons, findings are viewed more as hypothesis-generating than as hypothesis-driven. The results likely depended on the measures administered and on sample characteristics. Assessments were comprehensive, but some aspects of readiness, such as children’s health status, physical development, and temperament, were not evaluated. The present study also did not consider the level of support provided by families and communities as additional contributors to school readiness [8,9].

Data from all children who participated in the study were included in the analysis, and the VPT group was similar to other VPT cohorts in terms of mean GA and neonatal complications [31]. However, recruitment did not ensure representativeness relative to the broader VPT population. Findings may be more informative of the nature of readiness problems in preschoolers capable of engaging in testing than of the outcomes of VPT birth more generally. Additionally, FT preschoolers were recruited from a pool of families that had volunteered for research and that had higher mean zSES than the families of VPT preschoolers. Although zSES was controlled in analyses, the VPT and FT groups were not matched on background characteristics and it is unclear how representative the FT group was of the broader regional community of preschool children.

Evidence from LCA of distinct profiles of readiness is also preliminary and requires replication with larger samples. Samples larger than the present one are commonly recommended in conducting LCA, though smaller samples may be needed in LCA involving more and higher-quality indicators [66,67]. The substantial number of readiness measures assessed in this study may thus have contributed to the reasonable fit indices obtained by the present LCA. The four profiles of readiness characteristics identified by the LCA are also similar to those reported in previous studies of older VPT children. Additional studies are nonetheless needed to further our understanding of the nature of individual differences in readiness among VPT preschoolers. Examination of associations of profile types with subsequent academic progress and with medical and environmental risk factors would also be useful, as has been conducted in slightly older samples of VPT children [9,26,27].

Despite these limitations, the results have important clinical implications for assessing, monitoring, and treating readiness difficulties in VPT preschoolers. The sensitivity of methods for identifying readiness problems would be enhanced by assessing the aspects of readiness most likely to be affected by VPT birth. Based on the present findings, these domains include performance on tests of spatial and nonverbal cognition, executive function, motor skills, phonological processing, and numeracy. Findings also support the inclusion of caregiver ratings of dysexecutive behavior and problems in social functioning and emotion regulation. Readiness problems may be manifest in global weaknesses in test performance and psychosocial adjustment, or may manifest as milder or more selective deficits. Inclusion of tests of motor skills and measures of social functioning and emotion regulation appears particularly important in designing assessments of readiness, given the evidence for weaknesses in these areas that are independent of global cognitive ability.

Weaknesses in multiple readiness measures in 70% of the VPT group underscore the critical need for universal monitoring of readiness skills in this population before school entry [5,10,15,68]. Beyond surveillance, results of monitoring assessments allow efficient and specific targeting of interventions to address aspects of readiness most impaired in VPT preschoolers. These approaches might entail efforts to remediate skill deficiencies or to accommodate for them in ways that minimize disruptions to the learning process. The need for broader-based preschool interventions is underscored by VPT preschoolers’ difficulties in multiple readiness domains, along with the potential negative consequences of delayed readiness on longer-term educational and employment outcomes [4,5,10,19]. Exemplary interventions are programs that involve both school- and family-based activities and that address children’s socioemotional functioning, as well as their specific weaknesses in cognitive, motor, and early literacy and numeracy skills [13,19]. As similar proportions of the VPT and FT groups were enrolled in preschool, the present findings align with previous evidence suggesting that preschool attendance alone, absent interventions directed to the specific needs of VPT children, is unlikely to narrow the “preschool readiness gap” [69].

Follow-up of the present sample into early school age would be informative in investigating readiness measures and profiles as predictors of school-age achievement. Although associations of readiness measures with later achievement in both VPT preschoolers and larger community samples are well-documented [4,19,23,24], further research is required to identify the best predictors. Evidence that certain measures of readiness are highly predictive of subsequent achievement would argue for their inclusion in preschool assessments. A better understanding of the sources of readiness problems will also require more research on the neonatal, early developmental, and environmental factors associated with these deficits. The present findings are consistent with previous research in documenting associations of greater sociodemographic disadvantage with difficulties on several of the readiness measures [5,17,63,70]. However, more emphasis on the relation of readiness to other, potentially modifiable, characteristics of the family or preschool setting would have greater utility in developing interventions.

## 5. Conclusions

The findings from this study suggest that effects of VPT birth on school readiness are most likely to be manifest in tests of spatial, nonverbal, and motor skills, executive function, phonological processing, and early number knowledge. Adverse effects on behavioral aspects of readiness, such as behavioral manifestations of executive deficits and problems in social functioning and emotion regulation, are also evident, but are less pronounced or related to sex. Variability in the types of readiness problems displayed by VPT preschoolers is supported by findings of independent effects of VPT birth on global cognition and motor skills and by variable profiles of readiness skills in the VPT group. These results will help guide the development of more effective approaches to identify those VPT children in need of more intensive early educational interventions and may also help target those interventions. Efforts to replicate the present findings with larger and more representative samples are critical in refining methods for identifying individual variations in readiness and informing interventions targeted to specific profiles of strengths and weaknesses.

## Figures and Tables

**Table 1:** Sample characteristics.

Characteristic	Group	
	VPT (*n* = 53)	FT (*n* = 38)
Neonatal and early developmental status:		
Child age, mean (SD)	4.7 (0.29)	4.6 (0.29)
Gestational age, mean in weeks (SD, range) ^a,b^	27.3 (1.9, 23–30)	39.1 (1.1, 37–41)
Birth weight in grams, mean (SD, range) ^a,b^	944 (270, 369–1644)	3249 (435, 2325–4167)
Multiple birth ^c^, *n* (%)	10 (45)	2 (11)
Medical complications		
Grade III-IVH or PVHI ^d^, *n* (%)	6 (11.32)	-
Periventricular leukomalacia ^e^, *n* (%)	10 (18.9)	-
Bronchopulmonary dysplasia requiring home oxygen, *n* (%)	16 (30.2)	-
Intrauterine growth restriction (IUGR), *n* (%)	7 (13.2)	-
Culture-positive sepsis, *n* (%)	14 (26.4)	-
Necrotizing enterocolitis (NEC) ^f^, *n* (%)	6 (11.3)	-
Retinopathy of prematurity (ROP) requiring laser surgery, *n* (%)	4 (7.5)	-
Family demographic characteristics:		
Child sex: male, *n* (%)	27 (50.9)	18 (47.4)
Child race:		
White, *n* (%)	35 (66)	28 (73.7)
Black/African American, *n* (%)	10 (18.9)	3 (7.9)
Asian, *n* (%)	1 (1.9)	1 (2.6)
More than one race, *n* (%)	7 (13.2)	6 (15.8)
Child enrolled in preschool, *n* (%)	46 (86.8)	32 (84.2)
zSES Composite, mean (SD) ^a^	−0.25 (0.73)	0.35 (0.59)

Note. VPT = very preterm; FT = full term; IVH = intraventricular hemorrhage; PVHI = periventricular hemorrhagic infarction; zSES = sample z score composite of socioeconomic status. ^a^ Significant difference between groups, *p* < 0.05. ^b^ Data on these variables obtained from the medical record for all VPT children. These data were also obtained from the medical record for a majority of the FT group and from parent report when the medical record was not accessible. ^c^ Because several of the children were members of multiples and one was a sibling pair, a total of 39 families with VPT children and 36 with FT children participated in the study. ^d^ Grade III IVH is defined as hemorrhage with ventricular dilation and PVHI as hemorrhagic infarction in the periventricular region [34,35]. ^e^ PVL describes white matter injury. Consistent with other studies [34], we defined PVL as echogenecities in white matter that did not resolve on subsequent imaging and/or cysts or other evidence of white matter injury as identified by a radiologist. ^f^ Defined as Bells stage II or above and/or requiring surgical intervention.

**Table 2:** Readiness measures.

Domain/Measure	Reference	Description	Score
Global cognitive ability (DAS-II):	[44]		Standard Scores
DAS-II General Conceptual Ability		Global ability composite	
DAS-II Verbal Ability		Verbal comprehension and naming	
DAS-II Nonverbal Ability		Nonverbal reasoning	
DAS-II Spatial Ability		Perceptual motor skills	
Executive function and processing speed:			
NIH Toolbox Dimensional Change Card Sort	[45]	Cognitive ability/rule shifting	T-score ^a^
DAS-II Recall of Digits—Forward	[44]	Verbal working memory	T-score
Zoo Game Go No Go Task ^b^	[38]	Attention and inhibition	Proportion correct
CTOPP-2 Rapid Symbolic Naming ^c^	[46]	Speed of naming	Standard score
Motor skills (MABC-2):	[47]		Scaled scores
Total		Composite of motor skills	
Manual Dexterity		Fine motor skills	
Balance		Gross motor balance	
Aiming and Catching		Eye–hand coordination	
Early literacy and numeracy:			
TOPEL Print Knowledge	[48,49]	Letter names and sounds	Standard score
TOPEL Phonological Awareness		Ability to identify phonemes	Standard score
DAS-II Early Number Concepts	[44]	Counting and math problem solving	T-score
Behavior ratings:			
Conners EC-P Total Problems, Inattention/Hyperactivity, Anxiety, Social Functioning	[36,42]	Symptoms of overall problems and in areas of attention, externalizing, and socialization	T-scores
BRIEF-P Global Executive Composite, Inhibitory Self-Control Index, Flexibility Index, Emergent Metacognition Index	[50]	Behavior symptoms of executive dysfunction	T-scores
ERC Lability/Negativity, Emotion Regulation	[39]	Symptoms of mood lability and ability to regulate emotions	Raw scores

Note. DAS-II = Differential Ability Scales, 2nd Edition; CTOPP-2 = Children’s Test of Phonological Processing, 2nd Edition; MABC-2 = Movement Assessment Battery for Children, 2nd Edition; TOPEL = Test of Preschool Early Literacy; Conners EC-P = Conners Early Childhood—Parent; BRIEF-P = Behavior Rating Inventory of Executive Function-Preschool Version; ERC = Emotion Regulation Checklist. ^a^ Age-adjusted T-score. ^b^ The Zoo Go No Go Test was administered via computer during collection of evoked response potential data. The task served to index and time a series of trials as they appeared in the EEG stream, though only test performance was considered in this study. ^c^ For children with scorable performance on both Rapid Color Naming and Rapid Object Naming, CTOPP-2 Rapid Symbolic Naming was the composite of these two subtests. For the several children who were unable to complete one of these subtests due to lack of knowledge of color or object names, the Total score was based on the score for the completed subtest.

**Table 3:** Group differences in readiness measures.

	VPT Group	FT Group			
School Readiness Measure	M (SE)	M (SE)	β	*p*	Cohen’s d
Global/cognitive ability (DAS-II):					
General Conceptual Ability	92.17 (1.88)	108.97 (2.08)	−16.80	<0.001	−1.29
Verbal Ability	97.17 (1.97)	107.12 (2.21)	−9.95	0.002	−0.72
Nonverbal Ability	95.63 (2.02)	108.29 (2.22)	−12.66	<0.001	−0.92
Spatial Ability	90.08 (1.88)	106.03 (2.05)	−15.95	<0.001	−1.23
Executive function and processing speed:					
DCCS Age Corrected	94.53 (2.15)	100.05 (2.10)	−5.52	0.078	−0.42
DAS-II Recall of Digits-Forward	43.94 (1.77)	56.75 (1.98)	−12.81	<0.001	−1.22
Go No Go	0.62 (0.03)	0.75 (0.03)	−0.13	0.003	−0.70
CTOPP−2 Rapid Symbolic Naming ^a^	96.15 (2.80)	105.67 (2.77)	−16.68	0.031	−0.57
Motor skills (MABC−2):					
Total	5.52 (0.42)	8.91 (0.45)	−3.39	<0.001	−1.18
Manual Dexterity	4.90 (0.42)	8.29 (0.47)	−3.40	<0.001	−1.16
Balance	6.92 (0.40)	9.61 (0.46)	−2.69	<0.001	−0.96
Aiming and Catching ^b^	8.59 (0.43)	10.46 (0.51)	1.93	0.043	−0.79
Early literacy and numeracy:					
TOPEL Print Knowledge	96.80 (2.22)	107.26 (2.39)	−10.46	0.003	−0.69
TOPEL Phonological Awareness	92.37 (2.08)	106.53 (2.31)	−14.16	<0.001	−1.00
DAS-II Early Number Concepts	45.29 (1.27)	55.89 (1.49)	−10.60	<0.001	−1.31
Behavior ratings:					
Conners EC-P Global Index Total	56.23 (1.69)	55.23 (1.88)	1.00	0.702	0.09
Conners EC-P Inattention/Hyperactivity	55.81 (1.72)	56.42 (1.97)	−0.61	0.822	−0.05
Conners EC-P Anxiety	56.88 (1.74)	55.46 (1.93)	1.42	0.598	0.13
Conners EC-P Social Functioning ^c^	53.73 (1.29)	47.68 (1.48)	−12.61	0.001	0.64
BRIEF-P GEC	58.55 (1.66)	51.31 (1.91)	7.24	0.007	0.61
BRIEF-P Inhibitory Self-Control Index	56.60 (1.76)	51.89 (2.00)	4.71	0.092	0.38
BRIEF-P Flexibility Index	54.67 (1.53)	50.04 (1.73)	4.63	0.056	0.43
BRIEF-P Emergent Metacognition Index	60.18 (1.59)	51.42 (1.91)	8.77	0.001	0.76
ERC Lability/Negativity	27.74 (0.95)	26.58 (1.10)	1.15	0.447	0.17
ERC Emotion Regulation ^d^	26.31 (0.40)	28.39 (0.48)	3.57	0.003	−0.60

Note. VPT = very preterm; FT = full term; M (SE) = mean (standard error); DAS-II = Differential Ability Scales, 2nd Edition; DCCS = Dimensional Change Card Sort; CTOPP-2 = Children’s Test of Phonological Processing, 2nd Edition; MABC-2 = Movement Assessment Battery for Children, 2nd Edition; TOPEL = Test of Preschool Early Literacy; Conners EC-P = Conners Early Childhood Parent rating; BRIEF-P = Behavior Rating Inventory of Executive Function—Preschool version; GEC = Global Executive Composite; ERC = Emotion Regulation Checklist. ^a^ Group × sex interaction significant (*p* = 0.047); significantly lower scores on CTOPP-2 Rapid Symbolic Naming for VPT group compared to FT group only among males (*p* = 0.003, EF = −1.12). ^b^ Group × zSES interaction significant (*p* = 0.050); more pronounced weakness on MABC-2 Aiming and Catching for VPT group relative to FT group at lower levels of zSES. ^c^ Group × sex interaction significant (*p* = 0.001); significantly higher ratings of problems in social functioning for VPT group relative to FT group only among females (*p* < 0.001, EF = 1.38). ^d^ Group × zSES interaction significant (*p* = 0.004); significantly lower ratings of self-regulation for VPT group relative to FT group only among females (*p* < 0.001, EF = −1.42).

**Table 4:** Group differences in rates of deficits in school readiness.

	VPT Group (*n* = 53)	FT Group (*n* = 38)		
Area of Deficit	*N* (%)	*N* (%)	*p*	OR (95% CI)
Global/cognitive ability (DAS-II):				
General Conceptual Ability	16 (31)	0 (0)	0.017	31.85 (1.85, 548.40)
Verbal Ability	9 (18)	0 (0)	0.037	20.84 (1.21, 358.86)
Nonverbal Ability	10 (20)	1 (3)	0.021	10.96 (1.43, 83.82)
Spatial Ability	20 (42)	1 (3)	0.014	14.10 (1.73, 115.11)
Executive function and processing speed:				
DCCS Age Corrected	11 (26)	3 (8)	0.040	5.00 (1.07, 23.29)
DAS-II Recall of Digits, Forward	14 (28)	1 (3)	0.007	15.43 (2.09, 113.90)
Go No Go	16 (37)	2 (6)	0.013	6.67 (1.49, 29.86)
CTOPP-2 Rapid Symbolic Naming	6 (18)	3 (9)	0.654	1.39 (0.33, 5.91)
Motor skills (MABC-2):				
Total	33 (62)	6 (16)	<0.001	1.61 (1.31, 1.98)
Manual Dexterity	39 (74)	9 (24)	<0.001	1.58 (1.27, 1.97)
Balance	18 (34)	6 (16)	0.050	1.22 (1.00, 1.48)
Aiming and Catching	17 (32)	2 (5)	0.003	1.25 (1.08, 1.45)
Early literacy and numeracy:				
TOPEL Print Knowledge	14 (28)	1 (3)	0.043	10.08 (1.08, 94.43)
TOPEL Phonological Awareness	13 (26)	0 (0)	0.031	22.87 (1.33, 394.68)
DAS-II Early Number Concepts	12 (24)	0 (0)	0.039	20.00 (1.17, 342.69)
Behavior problems:				
Conners EC-P Global Index Total	16 (30)	8 (21)	0.888	0.92 (0.28, 3.04)
Conners EC-P Inattention/Hyperactivity	20 (38)	10 (26)	0.666	1.25 (0.46, 3.38)
Conners EC-P Anxiety	19 (36)	9 (24)	0.442	1.53 (0.52, 4.47)
Conners EC-P Social Functioning	17 (32)	1 (3)	0.033	14.77 (1.24, 176.29)
BRIEF-P GEC	28 (53)	4 (11)	0.001	6.90 (2.10, 22.72)
BRIEF-P Inhibitory Self-Control Index	25 (47)	6 (16)	0.039	3.34 (1.07, 10.48)
BRIEF-P Flexibility Index	18 (34)	4 (11)	0.146	2.63 (0.71, 9.69)
BRIEF-P Emergent Metacognition Index	30 (57)	3 (8)	<0.001	12.81 (3.70, 44.33)
ERC Lability/Negativity	12 (23)	5 (13)	0.577	1.45 (0.39, 5.42)
ERC Emotion Regulation	13 (25)	1 (3)	0.002	1.22 (1.08, 1.39)
Multiple Deficits:	44 (83)	12 (32)	0.001	1.42 (1.15, 1.76)

Note. VPT = very preterm; FT = full term; OR (95% CI) = odds ratio (95% confidence interval); DAS-II = Differential Ability Scales, 2nd Edition; DCCS = Dimensional Change Card Sort; CTOPP-2 = Children’s Test of Phonological Processing, 2nd Edition; MABC-2 = Movement Assessment Battery for Children, 2nd Edition; TOPEL = Test of Preschool Early Literacy; Conners EC-P = Conners Early Childhood Parent version; BRIEF-P = Behavior Rating Inventory of Executive Function—Preschool version; GEC = Global Executive Composite; ERC = Emotion Regulation Checklist.

**Table 5:** Model fit statistics for latent class analysis (LCA).

Fit Statistics	Class 1	Class 2	Class 3	Class 4	Class 5
AIC	8224.109	7972.723	7890.293	7843.928	7807.723
BIC	8298.268	8086.28	8043.248	8036.28	8039.472
SSA-BIC	8197.413	7931.845	7835.233	7774.686	7724.298
Entropy	-	0.944	0.963	0.966	0.958
LMR	-	285.385	116.43	80.365	65.277
*p*-value (LMR)	-	0.01	0.27	0.57	0.77
BLRT	-	285.385	116.43	80.365	65.277
*p*-value (BLRT)	-	<0.0001	<0.0001	<0.0001	<0.0001

Note. AIC = Akaike information criteria; BIC = Bayesian information criteria; SSA-BIC = Sample size adjusted BIC; LMR = Lo–Mendell–Rubin test; BLRT = bootstrap likelihood ratio test. Lower values on AIC, AIC, and adjusted BIC indicate better model fit. A significant *p*-value on the Vuong–Lo–Mendell–Rubin test, Lo–Mendel–Rubin test, or bootstrap likelihood ratio test indicate a better fit for the less parsimonious model.

## Data Availability

The data presented in this study are available from the corresponding author subject to approval by the investigators, institutional review, and an approved data use agreement. The data are not yet publicly available pending sufficient time for the investigators to report on the findings.
